# Application of Infrared Thermography in the Rehabilitation of Patients in Veterinary Medicine

**DOI:** 10.3390/ani14050696

**Published:** 2024-02-23

**Authors:** Alejandro Casas-Alvarado, Asahi Ogi, Dina Villanueva-García, Julio Martínez-Burnes, Ismael Hernández-Avalos, Adriana Olmos-Hernández, Patricia Mora-Medina, Adriana Domínguez-Oliva, Daniel Mota-Rojas

**Affiliations:** 1 PhD Program in Biological and Health Sciences, [Doctorado en Ciencias Biológicas y de la Salud], Universidad Autónoma Metropolitana (UAM), Mexico City 04960, Mexico; 2 Department of Neurobiology and Molecular Medicine, IRCCS Fondazione Stella Maris, 56128 Pisa, Italy; 3 Division of Neonatology, Hospital Infantil de México Federico Gómez, Mexico City 06720, Mexico; 4 Facultad de Medicina Veterinaria y Zootecnia, Universidad Autónoma de Tamaulipas, Victoria City 87000, Mexico; 5 Clinical Pharmacology and Veterinary Anesthesia, Biological Sciences Department, FESC, Universidad Nacional Autónoma de México (UNAM), Cuautitlán 54714, Mexico; 6 Division of Biotechnology—Bioterio and Experimental Surgery, Instituto Nacional de Rehabilitación-Luis Guillermo Ibarra Ibarra (INR-LGII), Mexico City 14389, Mexico; 7 Facultad de Estudios Superiores Cuautitlán, Universidad Nacional Autónoma de México (UNAM), Cuautitlán Izcalli 54714, Mexico; 8 Neurophysiology of Pain, Behavior and Assessment of Welfare in Domestic Animals, DPAA, Universidad Autónoma Metropolitana (UAM), Mexico City 04960, Mexico

**Keywords:** infrared thermography, rehabilitation, pain, thermal response, dermal perfusion

## Abstract

**Simple Summary:**

This review aims to analyze the application of IRT in the evaluation of gait, the recognition of lameness, the evaluation of the effectiveness of the therapy, and the superficial thermal response in veterinary medicine during the rehabilitation of patients. Due to the ability of infrared thermography to detect changes in the local temperature of several body regions, this tool might help identify and monitor the progress of musculoskeletal injuries in veterinary patients.

**Abstract:**

Infrared Thermography (IRT) has become an assistance tool in medicine and is used to noninvasively evaluate heat elimination during and after inflammatory processes or during the recovery period. However, its application in veterinary patients undergoing physiotherapy is a field that requires deep research. This review aims to analyze the application of IRT in the monitoring of animal physiotherapy, using the thermal changes that are present in patients undergoing gait or lameness issues (e.g., inflammation, pain, increased local temperature) as a neurobiological basis. Rehabilitation techniques such as acupuncture, physical therapies, thermotherapy, photo-biomodulation, and electrostimulation have been reported to have an anti-inflammatory effect that decreases the amount of local heat production, which is heat that can be recorded with IRT. Therefore, IRT could be used as a complementary tool to evaluate the effectiveness of the therapy, and it is suggested that further studies evaluate the accuracy, sensibility, and sensitivity of IRT.

## 1. Introduction

Infrared thermography (IRT) is a technique that uses a specialized camera to evaluate the infrared radiation related to the temperature of a body [1,2,3]. In mammals, the infrared radiation emitted from the body is highly related to the core body temperature and the ability of consequent changes in the peripheral blood flow to increase or decrease the amount of radiated heat [4,5,6]. IRT has been used to estimate the body temperature of animals and humans through surface temperature readings [7,8]. Nonetheless, several events can alter the blood flow of cutaneous blood vessels via shifts in peripheral vasodilation or vasoconstriction. Examples of these events are lesions in dermal or muscular tissue, and bone, ligament, joint, and tendon injuries [9,10,11,12]. After tissue damage, proinflammatory mediators are released, causing the vasodilation of capillaries, venules, or arterioles, with a subsequent increase in local temperature [13,14,15]. On the other hand, local hypothermia can be seen in patients with vascular compromise and ischemia issues [16,17].

For this reason, IRT has been suggested as a tool that can help to recognize injuries and changes in surface temperature and be implemented in the recovery process, particularly in cases requiring physical therapy [18,19,20,21]. Therapies such as acupuncture, photo biomodulation, electrostimulation, or thermotherapy can modify the superficial thermal response associated with the inflammatory process and pain [22,23,24]. Some studies have shown that, for example, electrostimulation with transcutaneous electrical nerve stimulation (TENS) prevents tissular necrosis [25], while high-intensity laser therapy applied to racehorses with back pain has been shown to decrease the palpation score for pain [26]. Although further studies are required, these events may show a distinct thermal response when evaluated through IRT [27,28,29]. This review aims to analyze the application of IRT to monitor animal physiotherapy, using the thermal changes that are present in veterinary patients undergoing gait or lameness issues (e.g., inflammation, pain, increased local temperature) as a neurobiological basis.

## 2. Search Methodology

A literature review was conducted using Web of Science, Scopus, Science Direct, and PubMed. The keywords used for the search were “animal rehabilitation”, “joint inflammation”, “physical therapy”, “electroacupuncture”, “thermotherapy”, “electrostimulation”, “acute pain” and “chronic pain”. The selected articles were studies or review papers published from 2000 to 2023 on animal rehabilitation techniques where IRT was applied as a complementary monitoring tool. Articles that only mentioned the application of the mentioned physical therapy techniques but did not consider IRT were excluded.

## 3. Thermal Response Associated with Physical Therapy

IRT is a method that directly evaluates the amount of heat radiation emitted from a physical body and, in mammals, is associated with thermoregulatory mechanisms [30,31]. In veterinary medicine, it has been used to identify superficial lesions due to the local inflammatory process taking place [9,13,32]. Most veterinary patients referred to physiotherapy have sustained inflammatory and painful conditions in the musculoskeletal system, hampering their quality of life.

One of the main objectives of physiotherapy is to relieve pain, restore mobility, or strengthen weakened muscles. Thus, IRT could help monitor the therapy’s progression and efficacy [33]. An example is the study by Coelho et al. [34], where acupuncture and laser needling were used to reduce nociception and inflammation in 140 male Swiss mice. They evaluated the superficial thermal response and the edema level in animals receiving acupuncture and laser puncture at sites LI11, ST36, GB34, and BL60. The surface temperature in the control animals was significantly higher than those receiving acupuncture or dexamethasone as an anti-inflammatory drug (control group = 31 °C vs. acupuncture or laser puncture = 26 °C). Likewise, both laser puncture and acupuncture reduced edema in the plantar region by 25%, equivalent to dexamethasone (35%). For the authors, these results demonstrate that acupuncture reduced inflammation and pain.

Other techniques, such as restorative and low-frequency magnetic therapy for five days, were studied in 25 adult Husky sled dogs [35]. By evaluating the surface temperature at the shoulder girdle muscle, this significantly increased after the therapy (up to 6.7 °C), but 1 h after the therapy session, the temperature in the region decreased by up to 5 °C, reaching a temperature of 30.1 °C after 10 min of magnetic therapy. The temperature drop was associated with reduced damage from injuries to the skeletal muscle tissue, a reduced inflammatory response, and superficial heat radiation. This coincides with what was reported by Parmen et al. [36] in New Zealand rabbits, in whom the effect of bipolar electroacupuncture for 20 min was analyzed. After analyzing repeated means, electroacupuncture significantly increased the surface temperature by 0.2 to 0.3 °C due to a local sympathetic modulation effect. In this regard, acupuncture is known to modulate the activity of the Autonomous Nervous System, which alters superficial blood flow through vasodilation events that can be reflected in a decrease in the surface temperature [37].

These results offer insight into the applicability of IRT in physiotherapy. However, additional research is needed to validate IRT as a tool able to monitor animal rehabilitation therapies.

## 4. Gait Evaluation and Lameness Recognition

In medicine, IRT has been used to evaluate musculoskeletal function due to the changes in the surface temperature caused by altered local blood flow [38,39,40]. This was reported by Repac et al. [41] when performing orthopedic and gait analyses in 11 healthy dogs after a 6 min walk. When comparing the before and after exercise, the surface temperature of the gastrocnemius and biceps femoris was significantly increased by up to 0.29 °C and 1.29 °C, respectively, due to increased vascularity.

Pichová et al. [42] also investigated the thermal changes in eight male German and Belgian Shepherd patrol guard dogs after a work routine. Thermal images were obtained bilaterally from 12 body regions, 5 min before and 30 min after training. A general increase in temperature was recorded at 5 and 30 min after activity, with average values of +1.24 °C and +0.9 °C, respectively, particularly in the limbs where a sustained increase was reported. The previous results coincide with what has been described in horses by Soroko et al. [43]. The neck, shoulder, chest, and croup temperature was monitored in Felin ponies during exercise on a treadmill. After five minutes of exercise, the temperature in all regions significantly increased by 1 °C, which was maintained for 20–25 min. Likewise, in the recovery period, the temperature of all regions decreased, particularly in the neck (from 33.22 to 32.80 °C) and chest (from 33.27 to 33 °C, respectively).

The mentioned studies suggest that physical activity and increased muscular metabolism also increase heat elimination after exercise. Nonetheless, the intensity of the thermal response might also be influenced by the type of muscular exercise. This was described by Farley et al. [44] in nine healthy dogs (three German Shepherds, and six Labradors) undergoing a 20 min treadmill exercise or rubble searches. Temperature differences were not detected for any muscle region in both types of activities. Moreover, both groups observed a muscle temperature reduction from pre to post exercise over the 14-week training period. An additional explanation for this lack of temperature difference may be due to the conditioning effect, which generates physiological and vascular changes that allow the hypertrophy of the cardiac muscle, which increases the efficiency of cardiac output and gas transport throughout the body, resulting in an increase in the physical capacity of the animal during strenuous exercises [45,46].

Regarding injured veterinary patients, Infernuso et al. [47] used IRT to differentiate between normal stifles and those with cranial cruciate ligament rupture (CCLR) in 16 Labrador Retriever dogs. When evaluating the stifle surface temperature of animals, it was found that the injured stifle was 1 °C above the healthy hindlimb. According to these results, the authors reported a success of 85% in the differentiation of normal stifles and those with CCLR in cranial, medial, caudal, and lateral images. Similarly, in dogs with ligament rupture, differences between normal and injured dogs were detected [48]. For CCLR patients, the injured stifle had an average temperature of 31.6–32.7 °C, while the normal stifle temperature was 27.1–29.2 °C. In canine cases of elbow dysplasia (Labrador, Golden Retriever, and Bernese dogs), thermal imaging performed in right/left forelimbs was able to correctly identify 100% of patients with abnormal elbows, recording differences of up to 0.9 °C, where abnormal elbows had the warmer values [49]. Another study applied IRT to differentiate between stifles with and without CCLR; however, Cain et al. [50] did not find significant differences in the maximum surface temperature of affected and unaffected stifles with CCLR in dogs of different breeds.

**Figure 1:**
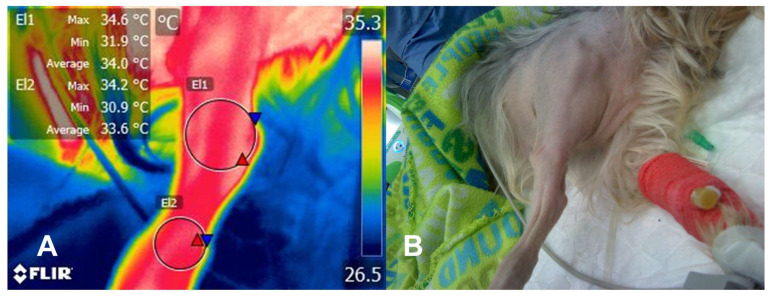
Thermal response in an inflammatory injury caused by a distal radioulnar fracture in a 2-year-old Yorkie Terrier. The radiometric image (**A**) of the left thoracic limb (**B**) with a radioulnar fracture shows that the maximum temperature (red triangle) at the fracture site (El1) was 34.6 °C, while the average and minimum values (blue triangle) were 34 °C and 31.9 °C, respectively. In contrast, the surface temperature of the radioulnar–carpal joint had lower temperatures, with an average difference of 1 °C. These differences between the temperature of the injured site and healthy tissue are related to the inflammatory process that causes the vasodilation of the capillaries close to the skin. These changes induce increased heat radiation, with the medium captured by the infrared camera.

The demonstrated evidence shows that IRT can help recognize injuries in joints or affected regions that present with an inflammatory process and might be associated with acute pain. Figure 1 shows the effect of the inflammatory response in a canine patient with a radioulnar fracture. Whittaker et al. [13] mention that during an inflammatory and chronic pain process, such as osteoarthritis (OA) or CCLR, immune cells such as neutrophils and macrophages release substances and chemicals such as interleukin (IL)-1, IL-10, IL-6, tumor necrosis factor-α (TNF-α) and prostaglandin F_2α_ (PGF_2α_); these are known as proinflammatory mediators since they increase local blood flow and, consequently, the surface temperature detected with IRT.

The usefulness of this tool in recognizing inflammatory lesions suggests that IRT is a technique able to assist patients with OA and aid in gait analysis. Alves et al. [51] used IRT to evaluate the response of working police dogs with bilateral hip OA. No significant correlations were found when comparing IRT with the Orthopedic Foundation for Animals hip grades. However, when comparing lateral vs. dorsoventral view images, lateral recordings had surface temperature that was higher by approximately 2 °C. Furthermore, a weak significant correlation was reported for the weight-bearing evaluation (r = 0.13, *p* < 0.01), the clinical metrology instruments scores (r = −0.25, *p* < 0.01), as well as the pain severity score when static (r = −0.25, *p* < 0.01) and during walking (r = −0.21, *p* = 0.04). In another study from the same authors on dogs suffering from the same condition, IRT evaluations at the lumbar region were related to Liverpool osteoarthritis in dogs and stiffness [52]. Nonetheless, other studies did not report an increase in the surface temperature of the stifle in dogs with OA, which had an average temperature of 31.6–32.6 °C while the control animals had a temperature of 32.8–34.3 °C [48].

A study conducted on 14 dogs with unilateral pelvic limb lameness and 14 healthy dogs compared and documented the changes in radiographic images of the pelvic limbs and related them to IRT. The researchers found that the thermal pattern of the animals with lameness allowed the successful differentiation of the affected limb in 88% of cases. Similarly, differences of 72.4% in the peak vertical force between the limping limb and the healthy one were found, indicating that the cut-off point was 41.77% (AUC = 0.93) [53]. These findings reaffirm that IRT can be used as a non-invasive method to recognize lameness and, together with other clinical tools, evaluate biomechanical issues.

## 5. Thermal Imaging as a Tool to Monitor the Treatment of Orthopedic Patients

**Figure 2:**
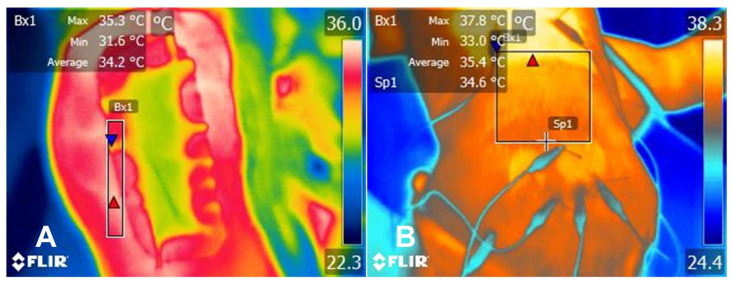
Comparison of the thermal response of a 5-year-old female Dash Hound during electroacupuncture. The dog presented with ambulatory paraplegia for 1 week and acute pain in the thoracolumbar region. After an advanced imaging study, spinal cord compression was observed in the 3–4° lumbar intervertebral space, requiring surgical intervention through laminectomy. (**A**) The local thermal response can be observed after laminectomy surgery, where the maximum temperature of the region of interest (Bx1) (red triangle) was 35.3 °C, the average was 34.2 °C, and the minimum (blue triangle) was 31.6 °C. (**B**) During electroacupuncture sessions, a significant increase in the maximum (red triangle) (+2.5 °C), average (+1.2 °C), and minimum (blue triangle) temperature (+1.6 °C) can be observed. This increase may be due to the sympathetic modulatory effect that acupuncture has, promoting vasodilation, increased heat radiation, and the release of endogenous opioids to diminish pain.

OA or CCLR patients do not only require techniques that enable a proper diagnosis to be reached, but treatment monitoring is also relevant [39]. Some studies suggest that evaluating the thermal response might indicate the effectiveness of the analgesic treatment administered to the animal [38,54]. Figure 2 shows the change in the thermal response that occurred in the superficial region of the injury site in the canine patient treated by the present authors and undergoing laminectomy due to disc compression, where IRT helped to recognize thermal changes after surgery and thoracolumbar pain. In contrast, Grossbard et al. [55] determined that, although IRT is a sensitive screening tool for dogs suspected of having thoracolumbar intervertebral disk disease (~90% success), thermal imaging alone cannot provide specific information regarding the type of lesion, and further studies are required to recommend IRT as a monitoring tool following surgical decompression.

Due to the response that may exist after rehabilitation therapy, IRT can also help in this area. This was observed in the study by Imboden et al. [56], which evaluated the analgesic effect of extracorporeal shock wave therapy on 16 horses with proximal suspensory desmitis or proximal suspensory desmitis-like pain in a hindlimb. After extracorporeal shock wave therapy, the gait analysis at 6, 24, 48, and 72 h was compared with the effect of the administration of local anesthesia. Despite finding a decrease in contralateral weight-bearing asymmetry 72 h after using extracorporeal shock wave therapy, they did not observe significant changes in post-treatment evaluation moments. The authors concluded that these results suggest that wave therapy has questionable effectiveness and requires further analysis.

Similarly, Mazzotta et al. [57] evaluated the variation in the body surface temperature of healthy dogs and those with spinal cord injuries after physiotherapy with a water treadmill in sixty-seven dogs of different sexes, breeds, body weights, and ages. The surface temperature of the spinal region with disc lesions was 1 °C higher than that of healthy animals. Interestingly, it was also found that water treadmill therapy influenced the surface temperature in the spine, where physical activity significantly increased the spinal temperature by 1.5 °C in healthy and injured dogs. In comparison, a decrease of 0.5 °C was observed after therapy. The lower temperatures suggested decreased tissue inflammation, promoted by physical exercise after physiotherapy.

These studies suggest a relationship between the decrease in the thermal response captured through IRT and the disease that affects the biomechanics of the animal [58,59]. Therefore, with this change in the surface thermal response, the objective effectiveness of the chosen therapy is likely associated with its clinical effectiveness. Collins [60] studied the effect of acupuncture on back pain in 36 dogs (24 dogs with pain and healthy dogs) and compared the IRT results with the pain scale scores. Animals with back problems received dry needle acupuncture at GV-14, BL-23 bilateral, Bai-hui, and Shen-shu for 15 min, while thermal images were collected in the back region. The authors found that the surface temperature after acupuncture treatment decreased by up to 1.60 ± 0.51 °F, a change that was greater in injured animals than in the control group (−0.44 ± 0.26 °F). These authors’ findings prove that the use of this therapy causes changes in local temperature in regions affected by acute pain and thus could be associated with decreased pain perception. This report coincides with some experimental and clinical data suggesting that acupuncture reduces the local inflammatory response and the thermal response [34,61].

In this sense, Huntingford and Pety [62] explain that acupuncture activates Hageman’s tissue factor XII, activating f prostaglandins and the degranulation of immune cells, which release endogenous opioids, a descending pain mitigating system [27]. The activation of these analgesic descending pathways helps to decrease pain perception and, in addition, inflammation. Lee et al. [63] evaluated the differences in the thermal response generated by analgesia with electroacupuncture and that generated by anesthesia with ketamine hydrochloride in nine dogs (five = ketamine and four = electroacupuncture with acupoints GV-5 and Bai-Hui). The authors observed that the administration of ketamine significantly decreased the temperature of the dorsocaudal, dorsocranial, ventrocranial, and ventrocaudal body regions by up to 1.5 °C after 30 min. This decrease was reversed with a significant increase in temperature by 2 °C after 90 min post treatment. In contrast, the animals that received electroacupuncture showed a significant increase of 1 °C in the surface temperature of the same regions after 30 min of treatment and a subsequent decrease of 0.5 °C at 90 min post treatment.

A possible explanation for this effect is the role of acupuncture plays in activating mast cell degranulation, which also releases substances such as histamine, proteases, heparin, and bradykinin. Consequently, the release of endogenous opioids also promotes local reactions, such as increased blood flow in the area and increased local immune responsiveness, which reduce inflammation and edema and facilitate pain relief [62,64]. Thus, this biphasic thermal response can be helpful for the clinician because the increase in the local immune response can help identify the effectiveness of this therapy.

Therefore, the effectiveness of therapies such as acupuncture causes changes in the local thermal response. Modifying the local thermal response makes it possible to associate this effect with the level of pain perception. Thus, this response could also help to complement pain evaluation methods objectively.

## 6. Infrared Thermal Monitoring during Rehabilitation Techniques

Based on the evidence already discussed, rehabilitation therapies have an impact on the local thermal response to physical activity [56,65]. This has been researched by authors such as Woo et al. [66], who evaluated the duration of cooling after cryotherapy on the skin over the stifle joints in 16 dogs after tibial plateau leveling osteotomy surgery. Although no significant differences in the average skin temperature in the medial view of the stifle were reported in the first 60 min, a decrease of 3–4 °C was observed during cryotherapy, which was followed by rewarming in the next 45 min. These results show that postoperative cryotherapy is an option that may be able to reduce postsurgical inflammation. Similarly, in canine cases of delayed recovery after tibial plateau leveling osteotomy, IRT has not only recorded significant increases in the front surface temperature of the affected joint (36.2 °C vs. 34.9 °C in a normal limb), but has also shown that the application of physiotherapy using a cold pack and TENS is able to alter the surface temperature around the stifle joint. After six sessions of physical therapy, the affected stifle joint had a maximum temperature of 35.4 °C, while the unaffected joint had a maximum temperature of 35.6 °C, showing no statistical differences between the limbs. Therefore, cryotherapy and electrostimulation reduce noninfectious inflammation, histamine release, joint edema, and muscle spasms [67].

In this sense, changes in vascularization have been accepted as an additional explanation for the modification of the local surface thermal response. This response was reflected in a study carried out by Villanova et al. [68], who evaluated the effect of two exercise protocols (for 30 s and 60 s) and different vibration levels (acceleration 1 g, 2.5 g and 5 g) on the stifle and rectal temperature in eleven mixed-breed adult dogs. In general, the authors observed that the average stifle temperature in all animals was 1.1 °C higher during the 30 s of exercise at different vibratory levels compared to the baseline moment. Furthermore, at 60 s, the highest temperature of 30.8 °C was observed when the vibration speed was set at 5 g. On the other hand, only a significant increase of 1.3 °C was observed in the rectal temperature after the 60 s of exercise. The authors explain that the increase in muscular and peripheral vascular activity on the joint is promoted by vibration via the maintenance of the muscle tone. A study by Agostinho et al. [69] using IRT, arterial Doppler ultrasound, and Doppler echocardiography in 16 healthy crossbreed dogs exposed to a single whole-body vibration at frequencies of 30 Hz, 40 Hz, and 50 Hz over 5 min with 10 min intervals of frequency exposure revealed no significant differences in the variables in the evaluation events. This reaffirms that the superficial thermal response is associated with the local microvascular response, which is modified in response to the therapies used [39].

Therefore, vasodilation or vasoconstriction modifies the superficial vasomotor response and the consequent heat elimination [30,70]. Moreover, alterations in the tissue perfusion may cause alterations in heat elimination. In this regard, Magnin et al. [71] evaluated the variations in the surface temperature and rapid hemodynamic changes in eight healthy piglets under general anesthesia exposed to different blood pressure levels. The surface temperature in the left forelimb was negatively correlated with the temperature gradient and blood pressure (r = −0.042), which led the authors to conclude that IRT is a tool able to detect early changes in peripheral perfusion. This is the main reason why it has been suggested that IRT can be used to evaluate and monitor perfusion recovery at the superficial level of wounds [38,39].

Some controlled experimental studies in rodents have reported that the alteration of controlled tissue perfusion causes a significant decrease in surface temperature in the affected tissue [72,73,74]. This has suggested that IRT could be used as a tool for evaluating the superficial thermal response to monitor wounds or injuries requiring treatment, such as flaps or tissue graft techniques [16]. This was reported in a clinical study of 16 cats (healthy cats = 10 and sick cats = 6). IRT was used to differentiate between non-ischemic conditions and animals with ischemic conditions such as aortic thromboembolism. The authors identified a decrease of 2.4 °C as a reference to differentiate between animals with thromboembolism from healthy animals with a sensitivity of 80% and a specificity of 100% [75].

Knowing that the superficial thermal response is modified using physical therapies for animal rehabilitation might serve as a set point at which to adopt IRT to monitor the improvement of muscular activity and tone [76,77,78]. Then, the change in the vasomotor response and superficial perfusion could be considered indicators of a response to rehabilitation therapy. Furthermore, the presence of pain, which is a sign of inflammation, could also be monitored through IRT. Nonetheless, it is necessary to validate the use of IRT and consider the elements discussed in the final section.

## 7. Perspectives and Limitations of Infrared Thermal Imaging

The discussed research shows that IRT might be adopted as a follow-up and assistance tool in rehabilitation, providing specific benefits. For example, IRT is a technique in which physical or chemical restraint is not required to assess the superficial thermal response. Most studies have performed thermal imaging during therapy sessions or clinical examinations without excessively handling the animals. This suggests that IRT can promote stress-free practices in veterinary medicine. When considering the neuroendocrine effect of stress on an animal’s homeostasis (e.g., hyperthermia), it is important to adopt techniques that might induce stress and alter the results [38].

However, some aspects still need to be considered in future studies. Although IRT monitors the local thermal response after physical therapies in orthopedic injuries where inflammatory processes are involved [47,68,79], an interesting perspective to explore is the possibility of applying this tool in physical therapies aimed at injuries to muscle tissue, where the response is possibly longer compared to joint injuries. In this sense, it is necessary to explore the effectiveness of the use of combined therapies, such as acupuncture or the use of vibrations, to reduce the level of pain [63,69], with a multimodal pain management approach that could help surgeons and veterinarians to decide whether the current analgesic protocol is adequate.

In this sense, assessing the ability of IRT to recognize not only the local increase in temperature due to an inflammatory process but also the presence of different pain degrees could help to increase the efficacy of the treatment. Pain recognition is a critical element implicit in animals undergoing rehabilitation therapies. Therefore, implementing IRT as a tool to monitor both muscular/joint injuries and progress, and the presence/absence of pain, might improve the prognosis and healing of animals [80].

Acknowledging some of the limitations of IRT is needed to guarantee the consistency of the thermal readings. Table 1 shows some of the technical specifications of IRT used by different authors and animal species, while Table 2 summarizes the elements that can alter the surface temperature of animals and need to be considered/controlled in future studies. Environmental elements such as direct solar radiation can increase the surface temperature to 0.56 °C, while a wind speed of 7 km/h or 12 km/h can reduce the surface temperature by 0.43 and 0.78 °C [81,82]. Moreover, to obtain accurate thermal readings, apart from considering these factors, adding a control group with the same or, at least, similar characteristics to the animals receiving physical therapy or treatment is necessary to validate IRT as an additional tool to monitor animal health [83]. Likewise, guaranteeing consistency in the delimited areas to assess thermal images could help reduce the variability found in future studies, particularly when referring to the fore or hindlimbs, where anatomical regions are used to define a muscular region or joint precisely.

On the other hand, due to the factors that influence the application of IRT (e.g., the presence of hair and its length), it would be advisable to evaluate the possibility of recognizing the thermal response in different thermal windows such as the lacrimal caruncle, ocular surface or other regions sensitive to sympathetic activation [6,88]. Therefore, it would be important to evaluate whether the attenuation of the thermal response in the injury site is consistent with the decrease in the response of the Autonomous Nervous System, which could be an adequate indicator of the effectiveness of rehabilitation therapy [89,90]. One of the most significant limitations of IRT is the need for knowledge regarding the reference values able to help recognize the thermal changes present at injury sites or the effect that hair presence might have on the thermal response. Therefore, it would be necessary to consider whether said response is affected if the injury site is shaved, which can modify the local thermal response.

**Table 2:** Factors that influence thermal imagining assessment.

Factor	Temperature Variation	Environment	Reference
Direct solar radiation	0.7–0.8 °C	Exterior	Barreto et al. [91]
Wind speed	0.43–0.78 °C	Exterior	Church et al. [81]
Distance from the object	0.03–1.2 °C	Interior or exterior	Faye et al. [92], Montmany and Tattersall [93]
Camera angle	0.8–2 °C	Interior	Montmany and Tattersall [93], Jiao et al. [94]
Hair length	0.3–0.5 °C	Interior or exterior	Nomura et al. [95]
Type of coat (hair, glabrous skin, feathers)	2–3.6 °C	Interior or exterior	Mota-Rojas et al. [96]

## 8. Conclusions

Limited studies in which IRT is adopted as a monitoring tool in veterinary patients undergoing physiotherapy have been published. However, it is known that veterinary patients with musculoskeletal injuries that require physical therapy undergo an inflammatory process that is associated with pain and lesions. Inflammation also involves a moderate increase in local temperature, a sign that IRT can monitor.

Rehabilitation techniques such as acupuncture, physical therapies, thermotherapy, photo-biomodulation, or electrostimulation have been shown to reduce the release of the pro-inflammatory substances that modify the local vasomotor response. Thus, although additional studies are required, assessing the local thermal response through IRT during physical therapy could help to monitor the recovery and progression of the veterinary patient, as well as represent a technique that could be applied in wound and local perfusion monitoring to improve and hasten the recovery of musculoskeletal injuries in veterinary patients.

## Figures and Tables

**Table 1:** Technical specifications of thermal imaging in rehabilitation studies performed in animals.

Camera Model	Distance from the Animals	Resolution (Pixels)	Ambient Temperature (°C)	Species	Sample Size	Statistical Analysis	Reference
AVIO TVS-200 EX	40 cm.	320 × 240	26	Dogs	6	ANOVA with Bonferroni	Valentini et al. [84]
FLIR T64Sc	1 m	n.s.	28	Horse	12	ANOVA with Tukey	Rodrigues et al. [85]
IRIS-5000	n.s.	n.s.	n.s.	Dogs	8	Paired *t*-test	Um et al. [61]
Thermal CAM	40 cm.	n.s.	21–25	Dogs	67	General linear model for repeated measures	Mazzotta et al. [57]
n.s.	1 m.	320 × 240	n.s.	Horse	41	Two-sided Wilcoxon signed-rank test	Dai et al. [86]
FLIR C2	40 cm.	320 × 240	n.s.	Mice	140	Two-way ANOVA with Student–Newman–Keuls	Coelho et al. [34]
IRIS-7.5	2.5–3 m	320 × 340	n.s.	Horse	10	ANOVA with Tukey	Edner et al. [87]

## Data Availability

Data sharing not applicable.
